# Should Steering Settings be Changed by the Driver or by the Vehicle Itself?

**DOI:** 10.1177/00187208221127944

**Published:** 2022-09-23

**Authors:** Timo Melman, Mark Weijerman, Joost de Winter, David Abbink

**Affiliations:** 2860 Delft University of Technology, Delft, Netherlands; Group Renault, Guyancourt, France; ENSTA ParisTech, Palaiseau Cedex, France; 2860 Delft University of Technology, Delft, Netherlands

**Keywords:** variable steering ratio, adaptive automation, adaptable automation, curve driving, driving simulator, function allocation

## Abstract

**Introduction:**

Cars are increasingly computerized, and vehicle settings such as steering gain (SG) can now be altered during driving. However, it is unknown whether transitions in SG should be adaptable (i.e., triggered by driver input) or adaptive (i.e., triggered automatically). We examined this question for road segments expected to require different SG.

**Objective:**

This paper aimed to investigate whether SG mode changes should be made by the driver or automatically.

**Methods:**

Twenty-four participants drove under four conditions in a simulator: fixed low gain (FL), fixed high gain (FH), a machine-initiated steering system, which switched between the two SG levels at predetermined locations (MI), and a driver-initiated steering system, in which the SG level could be changed by pressing a button on the steering wheel (DI).

**Results:**

Participants showed poorer lane-keeping and reported higher effort for FH compared to FL on straights, while the opposite held true on curved roads. On curved roads, the MI condition yielded better lane-keeping and lower subjective effort than the DI condition. However, a substantial portion of the drivers gave low preference rankings to the MI system.

**Conclusion:**

Drivers prefer and benefit from a steering system with a variable rather than fixed gain. Furthermore, although automatic SG transitions reduce effort, some drivers reject this concept.

**Application:**

As the state of technology advances, MI transitions are becoming increasingly feasible, but whether drivers would want to delegate their decision-making authority to a machine remains a moot point.

## INTRODUCTION

A vehicle’s steering gain (SG), also known as steering ratio or steering sensitivity, is a key parameter that determines how much the front wheels turn for a given steering wheel input (Gross, 1977; Reuter & Saal, 2017). Until the late 1990s, steering systems in production vehicles were designed with a fixed SG. A drawback of a fixed SG is that it cannot accommodate differences in desired sensitivity for different driving situations (Black et al., 2014; Jamson et al., 2007). If the SG is high, it may be more challenging to control the vehicle precisely when driving on a straight road at high speed (e.g., highway driving), as high control gains amplify motor noise (e.g., Chapanis & Kinkade, 1972; De Winter & De Groot, 2012). On the contrary, a low SG would enable more precise control but requires larger steering movements, which would be effortful when parking or driving through sharp curves (Kroes, 2019; Olson & Thompson, 1970; Reuter & Saal, 2017; Shoemaker et al., 1967).

Cars are becoming increasingly computerized, and vehicle settings that were once fixed can now be altered during driving (e.g., , De Winter et al., 2021; Shibahata, 2005). Two ways of implementing variable steering settings can be distinguished: changes can be initiated based on vehicle-state variables such as speed (e.g., Jamson et al., 2007; Millsap & Law, 1996; Shimizu et al., 1999) or as part of a driving mode, such as the sport mode (e.g., BMW, 2022; Koehn & Eckrich, 2004; Renault, 2022). However, a yet-unanswered question is whether changes in SG mode should ideally be initiated by the driver (e.g., via the press of a button) or automatically by the car. This question has important implications since literature suggests that large changes in SG may, in some cases, negatively impact driver safety and acceptance. In particular, in a study on lane changing, Russell et al. (2016) found that drivers needed several trials to get used to a new SG.

The effects of machine-initiated and human-initiated mode changes (also referred to as adaptive vs. adaptable automation) have previously been investigated in various human-automation interaction studies (e.g., Hancock, 2007; Li et al., 2013; Sauer et al., 2012). In a review, Kaber and Prinzel (2006) concluded that “*there is a substantial body of research on adaptive automation demonstrating performance and workload benefits over manual systems control and traditional, technology-centered approaches to automation. Unfortunately, the same cannot be said for adaptable systems …*”. In the same vein, it can be expected that driver-initiated SG changes will increase workload since there is an increase in the driver’s responsibility for system supervision. However, others have noted that human-initiated mode changes have benefits in terms of improving operators’ confidence and sense of control and reducing unpredictability (Kidwell et al., 2012; Miller & Parasuraman, 2007). It is also noted that machine-initiated mode changes will be ineffective if the triggers are inappropriate. Sauer et al. (2012), for example, found that performance-based mode changes yielded a higher workload than event-based mode changes and human-initiated mode changes. A possible explanation was that their performance-based trigger was not sensitive enough, resulting in infrequent adaptations (Sauer et al., 2012). In the same vein, Li et al. (2013) found that machine-initiated mode changes yielded a higher workload and lower preference ratings than human-initiated mode changes, as participants considered the triggering criteria confusing or inappropriate. In comparison, in the human-initiated mode-change condition, where operators were in charge of setting the level of automation, operators often selected the highest level of automation in which they had little to do.

The current study aimed to examine the effects of machine-initiated and driver-initiated changes in SG on lane-keeping performance, perceived effort, and system preference. Participants completed four conditions: fixed low SG (FL), fixed high SG (FH), machine-initiated steering that switched between the two SG at predetermined locations (MI), and driver-initiated steering in which the SG setting could be changed by pressing a button on the steering wheel (DI). It is noted that, technologically, the DI and MI concepts seem feasible on real roads, since these concepts require hardware such as steer-by-wire and a location-specific triggering mechanism. The latter is already part of intelligent speed assistance/adaptation (ISA), for example (Ryan, 2019).

To investigate MI and DI, three comparisons were prerequisites. First, FL was compared to FH to examine whether drivers indeed benefit from different SG in different parts of the road. More specifically, participants drove a route containing three driving-task segments—overtaking, driving on a straight road, and curve-driving—that were hypothesized to require different SG. For the overtaking and curve-driving segments, the FH condition was expected to produce more favorable outcomes (better lane-keeping, low effort) than FL, whereas for the straight segment, the opposite was expected. Second and third, the DI and MI conditions were compared with the “inappropriate” fixed-SG condition to examine whether the variable-SG conditions offer an overall benefit compared to FL and FH. Finally, we compared the MI and DI conditions, the primary topic of this work. Based on the above literature, it was expected that the MI condition would be less effortful for drivers than the DI condition, in which they had to change SG themselves.

## METHOD

### Participants

This research complied with the American Psychological Association Code of Ethics and was approved by the Human Research Ethics Committee of the TU Delft. Informed consent was obtained from each participant. Twenty-four participants (4 female) between 22 and 30 years old (*M* = 24.9, *SD* = 2.0) with a valid driving license and normal or corrected-to-normal vision participated in this study. In response to the question of how often they drove in the last 12 months, 1 participant drove less than once a month, 8 drove less than once a week, 13 drove 1–3 days a week, and 2 drove 4–6 days a week. Regarding mileage in the last 12 months, 6 participants drove 1–1000 km, 8 drove 1000–5000 km, 7 drove 5000–10000 km, 2 drove 10000–15000 km, and 1 drove 15000–20000 km.

### Apparatus

**Figure 1.**
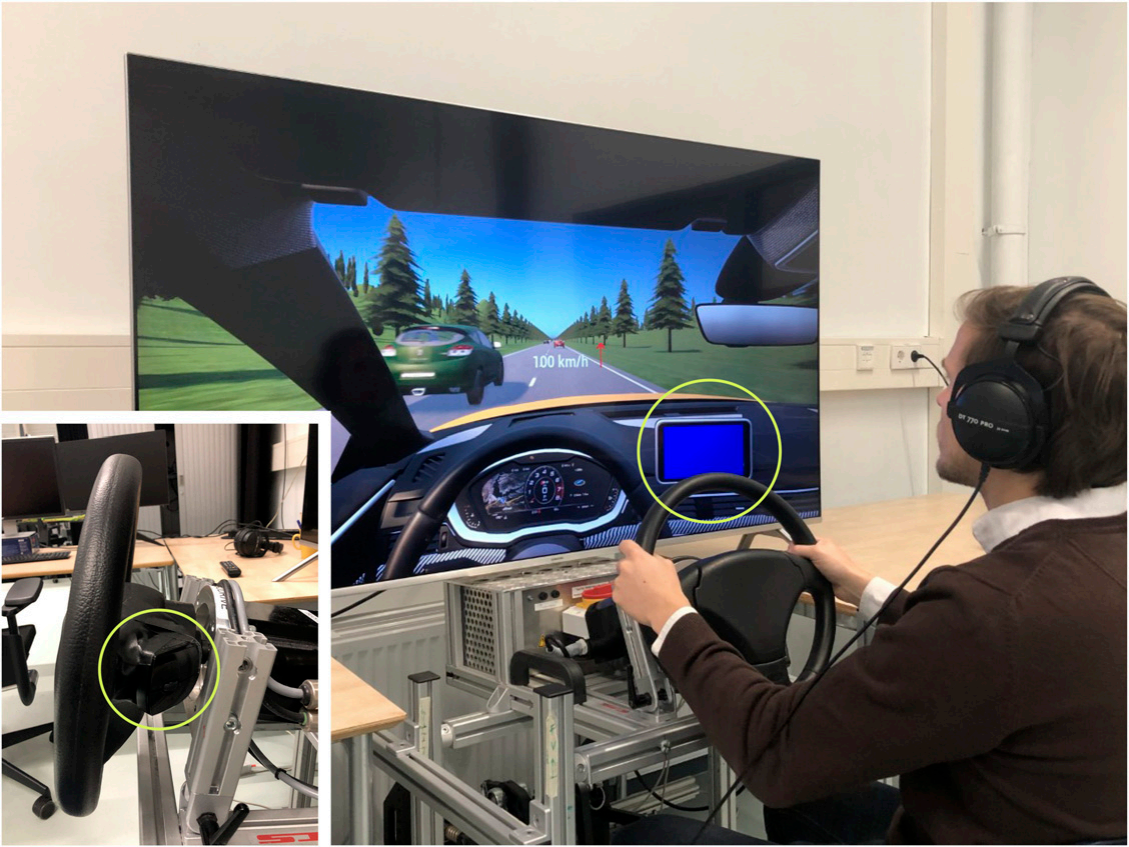
A participant driving in the driving simulator. The dashboard display shows the steering gain (SG) currently active (here colored blue, indicating low SG). The arrow next to the depicted speed indicates the advised SG level (available in the DI condition only). In this figure, participants are advised to switch “up,” from low SG to high SG. The left-bottom inset shows the button on the back of the steering wheel that was used to initiate the SG transitions. Participants wore headphones that displayed regular driving sounds “(Melman, Visser et al., 2021)”.

The experiment was conducted in a fixed-base driving simulator at the Cognitive Robotics laboratory at the Faculty of Mechanical Engineering of the Delft University of Technology. The simulation was developed using JOAN (Beckers et al., 2021), an open-source software framework developed at the Delft University of Technology, which builds on the CARLA open-source simulator (Version 0.9.8; Dosovitskiy et al., 2017). A 65-inch 4K screen was used to show the vehicle environment (Figure 1) with a refresh rate of 60 Hz. A SensoDrive® steering provided self-aligning torques with a fixed steering stiffness of 2.20 Nm/rad and a damping ratio of 0.60 Nms/rad. An Audi S4 (wheelbase 281 cm, width 185 cm, mass 1705 kg) was used to simulate the vehicle dynamics. The data was recorded at 100 Hz, and the update rate of the vehicle environment was 80 Hz. A mouse was attached to the back of the steering wheel for providing inputs to the driver-initiated steering system (left bottom corner Figure 1).

### Driving Tasks

All participants drove each trial on the same one-way two-lane road in one of the four conditions. The road was 14.3 km long, had 3.5 m wide lanes, and consisted of three segments of approximately equal length: an overtaking segment, followed by a straight segment and a curved road segment.

The participant’s car had a fixed speed of 100 km/h throughout each trial to ensure that the steering demands were the same for all participants. Driving is normally a self-paced task (De Winter et al., 2007; Taylor, 1964), and previous research in the same driving simulator showed that if drivers can choose their own speed, large individual differences in speed arise (Melman, Visser et al., 2021). These individual differences would complicate the comparisons between conditions because driving speed strongly affects lane-keeping performance (Godthelp et al., 1984).

In the overtaking segment, the participants, who drove at a constant speed of 100 km/h, had to swerve through traffic on a straight road. The other cars were driving 60 km/h and were alternately positioned in the left and right lanes (see Figure 1). Traffic density gradually increased from low (i.e., longitudinal spacing of 80 m, or 12.5 cars/km) to high (i.e., longitudinal spacing of 40 m, or 25 cars/km), and then gradually reduced back to low (longitudinal spacing of 80 m, or 12.5 cars/km). A higher traffic density can be expected to require a higher SG as the driver needs to provide faster steering inputs to fulfill the task. When the traffic density was high, the driver would have to make a lane change for every 100 m traveled, that is, every 3.6 seconds. In total, participants made 34 lane changes in the overtaking segment.

The second segment was a straight road without traffic, where the driver was instructed to stay in the right lane. The third and final segment consisted of a road with curves of different radii (between 100 and 200 m) without traffic.

### Independent Variables

All the participants drove in all of the following four conditions according to a counterbalanced within-subject design.

- Fixed low SG (FL)
- Fixed high SG (FH)
- Machine-initiated SG transitions (MI)
- Driver-initiated SG transitions (DI)

The FL condition featured a fixed steering ratio between the steering wheel and the front wheels of 25:1 throughout the entire track, whereas the FH condition used a fixed steering ratio of 12.5:1. These SG levels correspond to the literature. For example, Millsap and Law (1996) performed simulation studies with SGs of 24:1 and 14:1, where the latter was regarded as “*consistent with a vehicle that is perceived by drivers to be ‘darty’ or difficult to maintain directional control during highway driving*” (p. 1157).

The MI condition automatically switched between the two SG levels at predefined locations. During the overtaking segment, the settings changed from low SG to high SG when the traffic density became high (after 6 of 34 lane-change maneuvers) and from high SG to low SG when the traffic density became low again (after 28 of 34 lane-change maneuvers). Finally, 100 m before the curved road segment, the machine switched to high SG.

The DI condition allowed the driver to switch between low and high SG by clicking the left and right buttons of a horizontally-oriented mouse (see Figure 1). If the right (i.e., upper) mouse button was pressed, the steering system switched to high SG, and if the left (i.e., lower) mouse button was pressed, the steering system switched to low SG. All participants started the DI trial with the low SG setting. It was reasoned that to enable a meaningful comparison between the DI and MI conditions, participants in the DI condition should have access to the same knowledge about SG switches as available in the MI condition. Accordingly, participants in the DI condition were provided with switching advice, the locations of which were identical to the switching locations of the MI condition. The advised SG setting was displayed to the driver by an arrow: a downward facing arrow to suggest moving to low SG and an upward facing arrow to suggest moving to high SG (see Figure 1 for the upward arrow). If the driver already used the same steering setting as the machine would, no arrow was shown.

During a steering setting transition in the MI and DI conditions, the SG was linearly changed in 3.5 s. The current steering setting was visually communicated to the driver through a dashboard display which was either blue for low SG or red for high SG (Figure 1). The transition was visualized by changing the color of the dashboard display with a gradient between blue and red (from low to high SG) or between red and blue (high to low SG).

### Procedure

First, participants received a combined information sheet and consent form, which detailed the purpose, driving tasks, instructions, and procedures of the study. More specifically, the study was introduced as follows: “*the purpose of this study is to look into the effect of changing steering ratios initiated by the driver (you) or the machine itself. Two steering ratio settings (a slow steering and a fast steering mode) are tested in a machine-initiated steering system and a human-initiated steering system (where the driver can adapt the steering modes), and the designs are compared with two different passive steering systems (passive slow steering and passive fast steering). The effect of these systems is measured in terms of performance, safety margins, driver workload, and system acceptance.*” The document also mentioned the expected experiment duration of 1 hour, that the simulated car had a constant speed and only the steering wheel had to be controlled, that there were practice trials before each main trial, that participants had to complete four trials (FL, FH, MI, DI), and that each trial consisted of three segments (overtaking slow-driving vehicles, straight road without vehicles, curved road without vehicles). The consent form further explained that in the DI condition, participants could press the mouse buttons to change the SG level.

After reading and signing the informed consent form, participants were requested to sit in the simulator. They first drove two 2.5-minute familiarization trials, one trial with low SG and one trial with high SG, on a curvy road without other vehicles. The experiment was then started. Participants drove four trials, each trial in one of the four conditions (FL, FH, DI, or MI).

Before each experimental trial, a separate 2-minute practice trial was performed to let the participants experience the upcoming condition. The practice trial consisted of two straight-road segments and two curved-road segments. In the case of the DI condition, participants were encouraged to switch between high SG and low SG, and it was mentioned that they could switch whenever they wanted and that the advice displayed could also be ignored. In the practice trial of the MI condition, the SG switched automatically to low for straights and to high for curves.

Through the information sheet, participants were instructed to drive as follows: “*During the real trials you are asked to drive as you normally would with the emphasis on safe and controlled driving. The test track consists of three sections i.e. overtaking traffic vehicles which have a constant speed on a straight road, following a straight road without traffic vehicles and following a curved road without traffic vehicles. After the experiment you are asked to fill out a questionnaire. … Task instructions: During the entire track drive as you normally would. You are expected to drive on the right lane unless the traffic situation requires you to drive on the left lane.*” After each trial, the participants stepped out of the simulator and completed a questionnaire about the trial that was just completed. Finally, at the end of the experiment, participants completed a questionnaire about their overall experiences and preferences. The experiment took approximately 75 minutes per participant.

### Dependent Measures

The steering wheel angle and steering wheel speed from the SensoDrive were filtered with a zero-phase 2nd-order Butterworth filter for the data analysis. Dependent measures were calculated per participant for the following three road segments: overtaking (the part where the traffic density was maximal, between a traveled distance of 1963 m and 3464 m), straight (between a traveled distance of 5500 m and 9618 m), and curves (between a traveled distance of 9758 and 14345 m).

- *Mean absolute front wheel angle (deg)* describes the variability in steering output, where a higher value was considered poorer lane-keeping behavior. The front-wheel angle is the output of the driver’s steering wheel input after the conversion of the steering gain.
- *Mean absolute lateral velocity (m/s).* This is a measure of lane-keeping behavior, where a high lateral velocity can be seen as indicative of having to provide extra input to keep the car on the track.
- *Range of lateral position (m).* This measure, which is defined as the maximum lateral position minus the minimum lateral position, is an index of lane-keeping performance. A higher range implies that the participant made larger lateral excursions and therefore exhibited less safe driving behavior.
- *High SG (0 to 1).* The proportion of time that was driven with high SG. This measure was always 0 (i.e., low SG) for the FL condition and always 1 (i.e., high SG) for the FH condition. For the MI condition, it was 1, 0, and 1, for the overtaking, straight, and curve segments, respectively. For the DI condition, participants could decide whether to drive with the low or high SG setting, and so the value could take any number from 0 to 1. Steering gain settings were considered from the moment the button was pressed, that is, the 3.5-s transition period was not taken into account in computing the *High SG* gain measure.

Additionally, the following measures were obtained from the self-report questionnaire after each trial:

- *Subjective effort per segment (1 to 7)* was used to quantify the perceived effort of the driver per segment. After each trial, the participant was asked to answer for each of the three segments the question “*During the test, it took me little effort to overtake the traffic vehicles (follow the lane on the straight road/follow the lane on the curved road)*,” coded on a seven-point scale from 1 (*Fully agree*), 4 (*Neutral*), to 7 (*Disagree*).
- *Subjective workload (0 to 100).* The NASA-TLX questionnaire was used to determine participant workload on six facets: mental demand, physical demand, temporal demand, performance, effort, and frustration (Hart & Staveland, 1988). The items were rated on a 21-point scale from *Very low/Perfect* to *Very high/Failure*, and the overall score was determined as the mean of the six items and converted to a scale from 0 to 100.

Finally, the following measures were extracted from the post-experiment questionnaire:

- Overall steering system ranking. The participants were asked “*Which steering system do you prefer? Rank the four systems from 1 to 4 (1 most, 4 least)*”. Each number could only be used once.
- “*When overtaking the traffic vehicles (driving on a straight road/driving on a curved road) I prefer the slow steering response*,” coded on a seven-point scale from 1 (*Fully agree*), 4 (*Neutral*), to 7 (*Disagree*).
- Driver-initiated versus machine-initiated preference. The participants were asked “*Do you prefer letting the machine change the steering modes or changing the steering modes yourself (MI vs HI)?*” with response options machine-initiated and human-initiated.

### Statistical Analysis

Mean differences between the experimental conditions were examined using paired-samples *t*-tests. A total of four paired comparisons per dependent measure were made. First, FL was compared with FH. For the overtaking and curve-driving segments, the FH condition was expected to yield more favorable outcomes (better lane-keeping, low effort) than FL, whereas for the straight segment, the opposite was expected. Second and third, the DI and MI conditions were compared with the “inappropriate” fixed-SG condition for that segment to examine whether the variable-SG conditions offer a benefit compared to a static SG. Thus, for the overtaking and curve-driving segments, the comparison of DI and MI was made with FL, whereas for the straight segment, the comparison was made with FH. Finally, the DI and MI conditions were compared with each other.

Within-subject effect sizes *d*_*z*_ were calculated according to Faul et al. (2007). A Bonferroni correction was applied, which means that the alpha value of 0.05 was reduced by a factor of four (i.e., alpha = .0125).

## RESULTS

**Figure 2.**
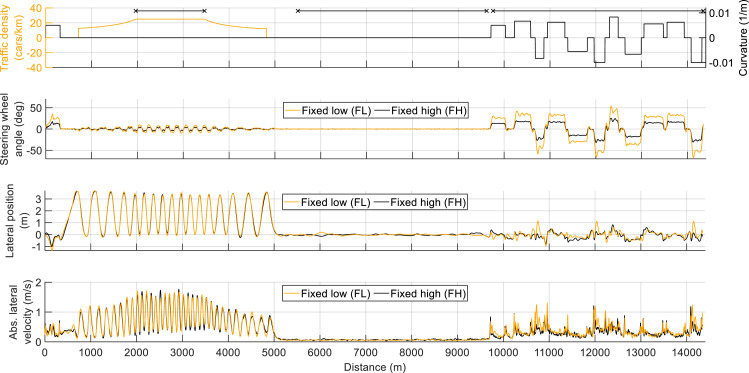
The mean steering wheel angle (second panel), mean lateral position with respect to the center of the right lane (third panel), and mean absolute lateral velocity (fourth panel) for the fixed low (FL) and fixed high (FH) conditions. Positive values indicate a left curve, steering, and lateral movement. The first (top) panel shows traffic density, road curvature, and three horizontal line segments that demarcate the three segments used in the analysis: overtaking, straight, and curves.

Figure 2 shows the mean steering wheel angle, mean lateral position, and mean absolute lateral velocity for the FL and FH conditions. The steering wheel angles in the FL condition were higher than those in the FH condition, due to the factor-two difference in SG. More specifically, the mean absolute steering wheel angle for FL and FH, respectively, was 7.33 and 3.84 deg in the overtaking segment, 0.41 and 0.30 deg in the straight segment, and 27.96 and 13.99 deg in the curve segment. For the curved road segment, some participants in the FL condition had difficulty driving through sharp curves, as reflected by large lateral excursions and high absolute lateral velocity.

**Figure 3.**
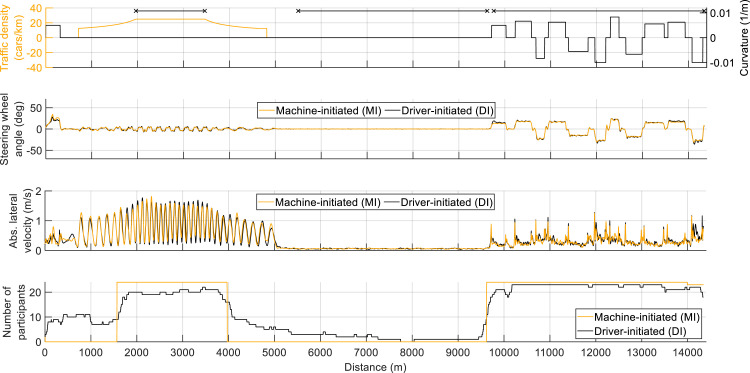
The mean steering wheel angle (second panel), mean absolute lateral velocity (third panel), and the number of participants driving with high steering gain (SG) (fourth panel) for the machine-initiated (MI) and driver-initiated (DI) conditions. Positive values indicate a left curve, steering, and lateral movement. The first (top) panel shows traffic density, road curvature, and three horizontal line segments that demarcate the three segments used in the analysis: Overtaking, straight, and curves.

Figure 3 shows the mean steering wheel angle, mean absolute lateral velocity, and number of participants driving with high SG for the MI and DI conditions. The steering angles were relatively similar for MI and DI, which can be explained by the fact that participants in the DI condition tended to follow the depicted advice and, accordingly, mostly drove with similar SG as in the MI condition.

However, as can be seen in the bottom panel of Figure 3, not all participants followed the advice, as some made intermediate switches. More specifically, in the MI condition, there were always three transitions between SG levels, while in the DI condition, the mean number of switches per participant was 5.50 (*SD* = 2.23, min = 3, max = 12). One participant drove the entire curve segment with low SG. It can also be seen that about five participants took a long time to switch back to low SG settings in the overtaking segment; that is, they appeared to have initially missed or ignored the presented advice and waited until they had overtaken all cars and drove on the straight, before switching to low SG. The switch back to high SG for the curve segment was more immediate, with about 10 participants even switching before the advice was displayed.

Table 1 shows the means, standard deviations, and effect sizes for the dependent measures per segment.

**TABLE 1:** Means, Standard Deviations, and Effect Sizes (*d*_*z*_) per Dependent Measure and Experimental Condition

	Mean				Standard deviation				Effect size (*d_z_*)					
	FL	FH	MI	DI	FL	FH	MI	DI	FL-FH	FL-MI	FL-DI	FH-MI	FH-DI	MI-DI
Overtaking segment														
Subjective effort (1 to 7)	2.54	1.96	2.25	1.83	1.50	1.00	1.48	1.24	0.42	0.16	0.40			0.22
Mean abs. front wheel angle (deg)	0.29	0.31	0.32	0.29	0.05	0.08	0.09	0.06	−0.22	−0.46	−0.03			0.41
Mean abs. lateral velocity (deg/s)	1.02	0.98	0.98	0.96	0.12	0.12	0.11	0.13	0.41	0.53	0.64			0.15
Lateral position range (m)	4.46	4.30	4.45	4.25	0.55	0.62	0.71	0.57	0.22	0.02	0.40			0.30
High SG (0 to 1)	0.00	1.00	1.00	0.83	0.00	0.00	0.00	0.31						
Straight segment														
Subjective effort (1 to 7)	1.42	2.21	1.46	1.63	0.78	1.50	0.66	0.92	−0.56			0.55	0.47	−0.30
Mean abs. front wheel angle (deg)	0.02	0.02	0.02	0.02	0.01	0.01	0.01	0.01	−0.77			0.85	0.63	−0.16
Mean abs. lateral velocity (deg/s)	0.06	0.07	0.06	0.06	0.02	0.03	0.02	0.02	−0.44			0.36	0.42	0.09
Lateral position range (m)	1.14	1.16	1.21	1.08	0.36	0.41	0.37	0.26	−0.06			−0.13	0.27	0.41
High SG (0 to 1)	0.00	1.00	0.00	0.07	0.00	0.00	0.00	0.14						
Curve segment														
Subjective effort (1 to 7)	3.75	2.71	2.29	2.92	1.48	1.23	0.81	1.28	0.73	1.14	0.67			−0.62
Mean abs. front wheel angle (deg)	1.12	1.12	1.12	1.12	0.01	0.01	0.02	0.02	−0.10	−0.30	−0.07			0.20
Mean abs. lateral velocity (deg/s)	0.37	0.32	0.31	0.33	0.11	0.08	0.07	0.09	0.95	1.01	0.66			−0.57
Lateral position range (m)	3.31	2.84	2.81	3.27	1.02	0.76	0.88	1.08	0.66	0.58	0.05			−0.60
High SG (0 to 1)	0.00	1.00	1.00	0.93	0.00	0.00	0.00	0.20						
Overall subjective ratings														
NASA TLX overall (0 to 100)	33.13	31.81	28.75	31.01	15.87	13.52	10.70	12.71	0.15	0.42	0.32	0.30	0.10	−0.30
Preference rank (1 to 4)	3.25	2.79	2.13	1.83	0.90	0.93	1.26	0.82	0.32	0.62	1.05	0.34	0.78	0.16

*Note:* *|d*_*z*_*|* > 0.553: *p* < .0125 (marked in boldface), *|d*_*z*_*|* > 0.769: *p* < .001. For the overtaking and curve segments, comparisons between DI/MI and FL are shown, and for the straight segment, comparisons between DI/MI and FH are shown (i.e., to test whether variable SG provides benefits compared to the fixed SG level that was deemed inappropriate for that road segment).

### FH vs. FL

In the curve-driving segment, participants’ subjective effort, mean absolute lateral velocity, and lateral position range were higher for FL than for FH. For the overtaking segment, differences between FL and FH were nonsignificant, but of the same sign. On the straight road, where high SG was expected to be detrimental, FH led to higher subjective effort than FL. Furthermore, the mean absolute front wheel angle was lower for FL compared to FH, suggesting that it was more difficult to drive accurately on a straight road with FH compared to FL. In summary, the comparison of FH and FL indicates that participants benefited from high SG in curves and from low SG on straights.

### MI & DI vs. FL/FH

As seen in Table 1, participants benefited from variable settings (MI & DI) in comparison to the fixed steering sensitivity. More specifically, on the straight segment, MI and DI yielded lower subjective effort than FH, and in the curve segment, MI and DI yielded lower subjective effort than FL. In the same vein, participants showed improved lane-keeping (lower lateral velocities during overtaking and curves, smaller absolute front wheel angles on straights) with MI and DI compared to the fixed SG levels.

### MI vs. DI

In the curve segment, a lower subjective effort was found for MI compared to DI. Also, participants showed poorer lane-keeping (larger range and velocity of lateral position) for the DI condition compared to the MI condition.

**Figure 4.**
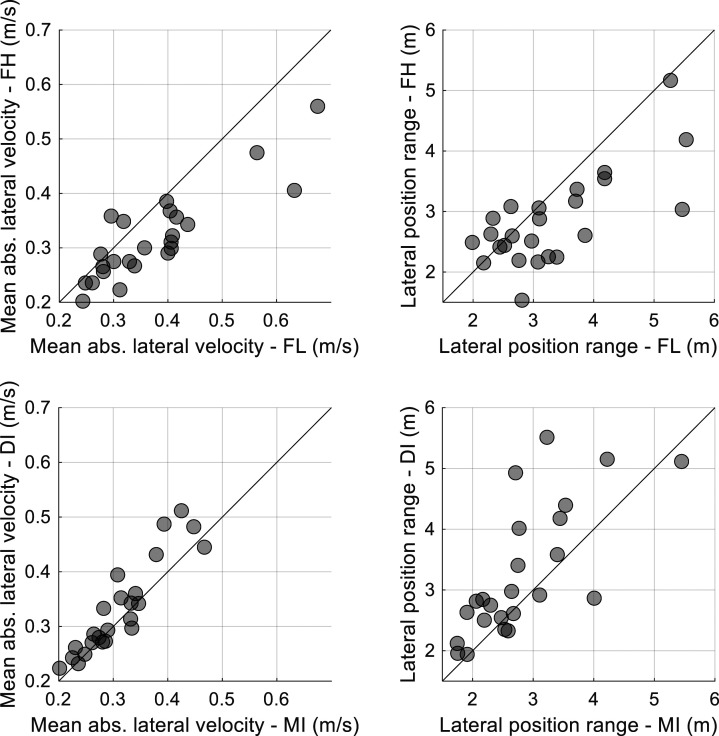
Mean absolute lateral velocity and lateral position range for the fixed high (FH) vs. fixed low (FL) conditions, and for the driver-initiated (DI) vs. machine-initiated (MI) conditions, for the curve-driving segment. Each marker represents a participant. The diagonal line is the line of unity.

Table 1 provides numerical information and does not elucidate how the experimental effects manifest themselves at the individual level. Therefore, a scatter plot is provided for several key comparisons of interest. More specifically, Figure 4 shows lane-keeping measures related to the curve-driving segment for FH versus FH (top two figures) and DI versus MI (bottom two figures). It can be seen that most points lie below or above the diagonal line, consistent with the statistically significant effects shown in Table 1. At the same time, individual differences are substantial, as could also be inferred from the large standard deviations in Table 1.

### Overall Ratings of Conditions

**Figure 5.**
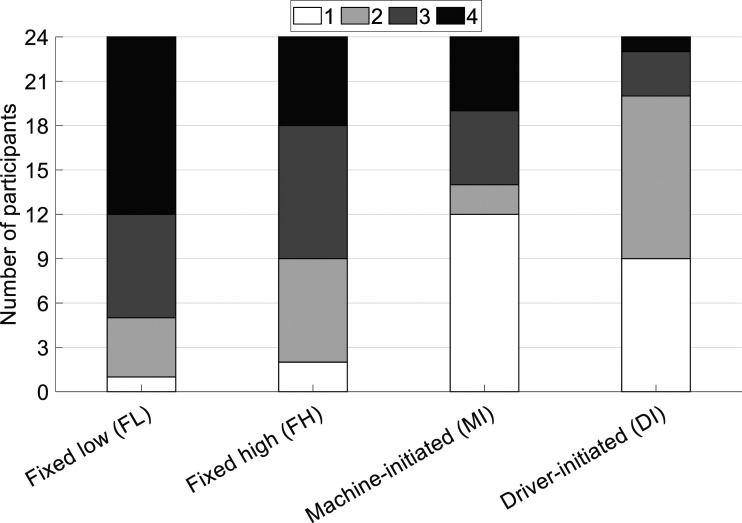
Ranking of the steering systems, with 1 representing the most preferred and 4 the least preferred.

The NASA-TLX showed the lowest workload for MI and the highest for FL; however, these effects were not statistically significant. The post-experiment ranking showed that MI and DI were significantly better ranked than FL, and DI better than FH. Interestingly, although 12 participants ranked MI as the most preferred, 10 participants ranked it third or fourth (Figure 5). In comparison, for the DI condition, 9 participants ranked it first, and only 4 participants ranked it third or fourth. To the question, “*Do you prefer letting the machine change the steering modes or changing the steering modes yourself?*”, 11 participants reported preferring MI, and 13 preferred DI.

Finally, in response to the question: “*I prefer the slow steering response*,” means (*SD*s) on the 7-point scale from 1 (*Fully agree*) to 7 (*Disagree*) were 4.54 (1.93), 1.38 (0.65), and 6.08 (0.97), for overtaking, straight-line driving, and curve-driving, respectively. These findings are consistent with the above results in that different driving environments require different SG levels.

## DISCUSSION

This study aimed to examine the effects of adaptive and adaptable transitions in steering gain (SG) on perceived effort, lane-keeping behavior, and system preference. In a simulator experiment, we compared two fixed SG levels and two systems that could switch between the low and high SG, either in an adaptable manner, that is, initiated by the driver via a press of a button (DI), or in an adaptive manner, that is, automatically by the car and triggered based on the location of the vehicle (MI). A test road was designed with segments that were hypothesized to require different SG settings. Based on the literature, we expected that machine-initiated transitions would reduce workload since the machine controls the adaptation and the driver is able to focus on the driving task.

In accordance with the intended experimental design, different SG levels were found to be appropriate for different road segments: Compared to a fixed low steering gain (FL), driving with a fixed high steering gain (FH) was perceived by participants as more effortful and resulted in poorer lane-keeping behavior on straights, while the opposite held in curves and to a lesser extent during overtaking maneuvers. A possible explanation for the relatively small differences between FL and FH in the overtaking segment is that the required steering angles were not as large as in the curve driving segment (see Figures 2 and 3, Table 1). That is, although high SG was the recommended setting for the overtaking segment, the overtaking segment could also be comfortably driven with low SG. Literature indicates that the relationship between control-output gain and task performance follows a U-curve that results from the benefits of high gain in terms of movement amplitude and benefits of low gain in terms of precise control (Chapanis & Kinkade, 1972; MacKenzie, 2013, p. 81). Our steering sensitivity levels were selected based on realistic values (Millsap & Law, 1996) and a pilot study (Kroes, 2019). The observed SG × road-type interaction suggests that drivers may benefit from a steering gain that is adjusted or adjustable. The experimental results concur that the MI and DI conditions yielded favorable outcomes compared to driving with the SG level that was inappropriate for that road segment.

The literature indicates that, compared to adaptable automation, adaptive automation reduces workload at the possible cost of unpredictability: “*Doing tasks directly costs more workload, but the payoff is greater awareness of how the task is being done*” (Miller & Parasuraman, 2007, p. 60). The current study also found a workload reduction for adaptive automation. More specifically, on curved roads, the MI condition produced lower effort ratings combined with better lane-keeping performance than the DI condition. The explanation for these findings is two-fold: (1) In the DI condition, there are at least some participants who drove with the “wrong” setting for some of the time (i.e., low SG instead of high SG), making them susceptible to the same performance deficits as observed in the FL condition, and (2) in the DI condition, drivers had to spend some effort to determine if and when a switch can be made and press a button (usually participants did so when driving in between curves).

In the overtaking segment, however, there were no significant differences in effort ratings between MI and DI. This lack of effort reduction for MI in the overtaking segment may be explained by unpredictability: the automatic SG switch occurred while the participants were still overtaking cars (in comparison, for the curve segment, the switch occurred 100 m before the first curve). This explanation is supported by the fact that some participants in the DI condition did not readily respond to the low SG advice after the overtaking segment, suggesting that this advice was ignored or missed. The ranking of the four conditions showed that the MI and DI steering systems were more preferred than the fixed steering gains. However, despite the improved performance for MI, a substantial portion of the participants gave low preference rankings to the MI condition. Possible explanations are that drivers in the MI condition disliked its unpredictability and the inability to choose the steering gain themselves. More generally, literature in aviation automation suggests that automation-mode confusions may arise if mode changes are not initiated by the human operator but by an external trigger (Sarter & Woods, 1995). Similarly, it can be expected that, despite the colored SG mode display on the dashboard, some drivers in the MI condition had difficulty understanding why an SG switch had occurred. Future research should examine whether the SG setting should be displayed to drivers, such as in the current study, or whether this information should remain hidden. The latter solution may have some benefits as it minimizes visual distraction, but it may also exacerbate mode confusion.

In comparison to the MI condition, the DI condition gave drivers flexibility. In essence, if drivers in the DI condition preferred low (high) SG, they could select the low (high) SG setting at the start of their drive. That is, the DI condition can deliver what FL and FH can also deliver, which can explain why the DI condition was hardly ranked third or fourth (see Figure 5). This observation is in line with a study on adaptable automation by Sauer and Chavaillaz (2018), which concluded that the primary advantage of adaptive automation is that it supports diverse types of operators, who differ in their preferences. A correlation analysis provided support for the notion that the DI condition facilitated individual preferences: participants who ranked the FL condition higher (i.e., more preferred) were more likely to select a low SG in the DI condition (see Supplementary Material). Even though MI delivered better driving performance than DI, it can be argued that driver preference is just as important, or as noted by De Waard and Brookhuis (1999): “*A system may function perfectly in the technical sense, if it is not accepted by the public, it will not be used*” (p. 50). An additional advantage of the DI system compared to the MI system is that the DI system is easier to implement, as it does not rely on GPS and maps that define which SG level should be selected.

As shown in Figure 3, participants in the DI condition drove with approximately the same SG level as in the MI condition. That is, the great majority of participants in the DI condition switched to high SG when overtaking cars, to low SG on the straight, and back to high SG in curves. The high similarity may be due to the fact that the trigger locations of the MI system were chosen appropriately, that is, in such a way that they correspond to the participants’ preferred SG setting. However, the high similarity could just as well be caused by the strong tendency of participants to follow the switching advice displayed in the DI condition. Possibly, participants’ reliance on the advice in the current experimental setting was stronger than it would be when driving a real car, where disuse of feedback systems is a known concern (Kidd et al., 2017). On the other hand, about half of the participants made the switch *before* the high-SG advice was displayed, suggesting that these participants anticipated the upcoming steering demands and were not just relying on the advice.

A limitation of our experiment was that it was conducted with young, and predominantly male students with a relatively low yearly driving mileage. Future research is needed with other population groups, such as expert and older drivers. Based on earlier literature on ADAS and older drivers (Classen et al., 2019; Young et al., 2017), it can be expected that older drivers may benefit from automation support, such as offered by the MI condition. At the same time, older drivers may have more difficulty driving while simultaneously processing other visual information, such as mode status and mode-changing advice.

Another limitation is that the machine-initiated transitions occurred at preprogrammed locations. Future studies could consider the current steering angle and driver state to determine safe moments for machine-initiated transitions. Note that in the current study, abrupt and potentially dangerous SG transitions were prevented by smoothly changing the SG over a period of 3.5 s. Future research should also examine whether our findings replicate for different types of HMIs and advice in the DI condition or no displayed advice at all, and conditions in which the driver can choose from a range of SG levels. Furthermore, future studies could explore the potential benefits of variable SG for specific situations, such as understeer or oversteer prevention (e.g., Heathershaw, 2004) and lane changes (e.g., Wang et al., 2017).

This study found that different driving tasks (e.g., overtaking and curve driving vs. straight-line driving) benefit from different SG levels and that a flexible SG (DI & MI) reduces subjective effort and yields better lane-keeping than a static SG (FL or FH). In turn, the MI condition yielded lower effort and better lane-keeping performance in curves than the DI condition, but was disliked by some drivers. Whether the same driving behaviors would be elicited on real roads, on which drivers can be expected to be ‘satisficers’ (Hancock, 1999), and speed is not kept constant, remains unknown. Although simulators have been found to exhibit relative validity in short-lasting experiments such as ours (e.g., Klüver et al., 2016), how drivers would respond to MI and DI systems in the long term is unknown. An analysis of learning trends showed that participants demonstrated slightly smaller steering angles as the experiment progressed, indicating more stable control (see Supplementary Material). With prolonged driving experience, underutilization and disuse of technology can become factors to be considered, as indicated above. Test track studies and field operational tests would be required to examine whether DI and MI are viable and safe solutions for future traffic.

## KEY POINTS

- Simulator study examined variable versus fixed steering gain (SG) on different road types
- Variable SG was machine-initiated (MI) or driver-initiated (DI)
- Variable SG yielded lower effort ratings and better lane-keeping than fixed SG
- On curved roads, MI yielded lower effort ratings and better lane-keeping than DI

## Supplemental Material

Supplemental Material - Should Steering Settings be Changed by the Driver or by the Vehicle Itself? Supplemental Material for Should Steering Settings be Changed by the Driver or by the Vehicle Itself? by Timo Melman, Mark Weijerman, Joost de Winter, and David Abbink in Human Factors
