# Supplementary material for: Should Steering Settings be Changed by the Driver or by the Vehicle Itself?
Source: Hum Factors. 2022 Sep 23;66(4):1201–15. doi: 10.1177/00187208221127944 (PMC10900860; doi:10.1177/00187208221127944)
Supplement: Supplemental Material - Should Steering Settings be Changed by the Driver or by the Vehicle Itself? [file sj-pdf-1-hfs-10.1177_00187208221127944.pdf]

Supplementary Material

Table S1. Means, standard deviations, and effect sizes (d_z) per dependent measure and order of presentation (1 = first trial, 2 = second trial, 3 = third trial, 4 = fourth trial).

	Mean				Standard deviation				Effect size (d_z)					
**Overtaking segment**	**1**	**2**	**3**	**4**	**1**	**2**	**3**	**4**	**1-2**	**1-3**	**1-4**	**2-3**	**2-4**	**3-5**
Subjective effort (1 to 7)	2.38	2.13	2.04	2.04	1.44	1.30	1.20	1.43	0.18	0.22	0.16	0.06	0.05	0.00
Mean abs. front wheel angle (deg)	0.32	0.30	0.30	0.29	0.05	0.08	0.07	0.09	0.30	0.44	0.39	0.08	0.18	0.13
Mean abs. lateral velocity (deg/s)	1.02	0.98	0.97	0.96	0.11	0.14	0.11	0.12	0.37	**0.70**	0.55	0.17	0.22	0.05
Lateral position range (m)	4.53	4.28	4.34	4.31	0.55	0.66	0.61	0.63	0.38	0.39	0.31	-0.12	-0.04	0.05
High SG (0 to 1)	0.46	0.45	0.48	0.45	0.49	0.50	0.50	0.50	0.01	-0.03	0.01	-0.04	0.00	0.04
**Straight segment**														
Subjective effort (1 to 7)	1.63	1.67	1.83	1.58	1.13	1.20	1.17	0.65	-0.04	-0.14	0.03	-0.17	0.08	0.24
Mean abs. front wheel angle (deg)	0.02	0.02	0.02	0.02	0.01	0.01	0.01	0.01	-0.01	0.18	-0.01	0.13	0.00	-0.07
Mean abs. lateral velocity (deg/s)	0.06	0.06	0.07	0.06	0.02	0.02	0.02	0.03	-0.23	-0.40	-0.16	-0.15	0.02	0.12
Lateral position range (m)	1.11	1.14	1.22	1.11	0.30	0.38	0.31	0.41	-0.09	-0.37	0.00	-0.26	0.09	0.29
High SG (0 to 1)	0.02	0.03	0.02	0.00	0.07	0.11	0.08	0.01	-0.04	-0.03	0.27	0.01	0.20	0.26
**Curve segment**														
Subjective effort (1 to 7)	3.33	2.96	2.79	2.58	1.31	1.12	1.41	1.38	0.26	0.31	0.43	0.12	0.30	0.22
Mean abs. front wheel angle (deg)	1.13	1.12	1.12	1.12	0.02	0.01	0.01	0.01	0.45	0.41	**0.71**	0.07	0.50	0.30
Mean abs. lateral velocity (deg/s)	0.33	0.33	0.34	0.34	0.09	0.08	0.10	0.10	0.12	-0.05	-0.13	-0.11	-0.27	-0.05
Lateral position range (m)	3.17	3.03	2.98	3.07	1.27	0.75	0.74	1.01	0.15	0.18	0.11	0.07	-0.07	-0.10
High SG (0 to 1)	0.49	0.45	0.50	0.50	0.50	0.50	0.51	0.51	0.04	-0.01	-0.01	-0.05	-0.05	0.00
**Overall subjective ratings**														
NASA TLX overall (0 to 100)	33.06	30.80	30.63	30.21	11.47	13.60	15.23	12.94	0.34	0.22	0.31	0.02	0.09	0.05
Preference rank (1 to 4)	2.50	2.46	2.25	2.79	1.14	0.93	1.33	1.06	0.03	0.11	-0.16	0.11	-0.20	-0.29

Note. The effect sizes are color-coded for visual clarity purposes. The color-coding ranges from -1 (red) to 0 (white) to 1 (green). $|d_z| > 0.553$: $p < .0125$ (marked in boldface), $|d_z| > 0.769$: $p < .001$.

Table S2. Means (M), standard deviations (SD), and correlations between the proportion of time driving with the high SG settings in the driver-initiated (DI) condition, and preference rankings for the four conditions ($n = 24$).

	M	*SD*	1	2	3	4	5	6
1. Preference rank FL (1 to 4)	3.25	0.90						
2. Preference rank FH (1 to 4)	2.79	0.93	-.25					
3. Preference rank MI (1 to 4)	2.13	1.26	-.37	-.57				
4. Preference rank DI (1 to 4)	1.83	0.82	-.24	.01	-.49			
5. High SG (0 to 1), DI overtaking	0.83	0.31	.27	-.38	.12	-.06		
6. High SG (0 to 1), DI straight	0.07	0.14	.26	-.29	-.10	.20	.11	
7. High SG (0 to 1), DI curves	0.93	0.20	.56	.00	-.34	-.09	.56	.00
